# Amine-functionalized copper-coordinated cryogels for efficient removal of Acid Blue 113 from water

**DOI:** 10.1038/s41598-026-47887-8

**Published:** 2026-04-12

**Authors:** Kadir Erol, Demet Tatar, Aysel Veyisoğlu, Gönül Arslan Akveran, İlknur Tosun Satır

**Affiliations:** 1 Vocational School of Health Services, Department of Environmental Protection Technologies, Hitit University, 19030 Çorum, Turkey; 2 Department of Medical Services and Techniques, Osmancık Ömer Derindere Vocational School, Hitit University, 19500 Çorum, Turkey; 3 Vocational School of Health Services, Department of Medical Services and Techniques, Sinop University, 57000 Sinop, Turkey; 4 Department of Food Processing, Alaca Avni Çelik Vocational School, Hitit University, 19600 Çorum, Turkey; 5 Department of Chemistry, Faculty of Engineering and Natural Sciences, Hitit University, 19030 Çorum, Turkey

**Keywords:** Acid Blue 113, Adsorption, Antibacterial activity, Cryogel, Dye removal, Chemistry, Environmental sciences, Materials science

## Abstract

A macroporous Poly(2-Hydroxyethyl methacrylate-glycidyl methacrylate) [Poly(HEMA-GMA)] cryogel functionalized with Poly(L-lysine) (PLL) and Cu(II) ions was developed for the efficient removal of the anionic dye Acid Blue 113 from water. The cryogel was synthesized via free-radical cryopolymerization of HEMA and GMA at − 20 °C, followed by covalent grafting of PLL through epoxy ring-opening reactions and subsequent coordination of Cu(II) ions with PLL amine groups. SEM analysis confirmed a highly interconnected macroporous structure, while BET analysis revealed specific surface areas ranging from 6.45 to 7.85 m^2^ g^−1^ and average pore diameters between 36 and 41 nm. Batch adsorption experiments demonstrated strong pH dependence, with maximum adsorption at pH 4.0. The Poly(HEMA-GMA)-PLL-Cu(II) cryogel exhibited a high adsorption capacity of 501.6 mg g^−1^ and reached equilibrium within 30 min, indicating rapid adsorption kinetics. Kinetic data were best described by the pseudo-second-order model, while equilibrium data fitted well to the Langmuir isotherm, suggesting monolayer adsorption on homogeneous active sites. Additionally, the Cu(II)-containing cryogel exhibited notable antibacterial activity against several Gram-positive and Gram-negative bacteria. These findings demonstrate that the developed cryogel combines high adsorption capacity, rapid kinetics, and antimicrobial functionality, making it a promising material for advanced wastewater treatment applications.

**Supplementary Information:**

The online version contains supplementary material available at 10.1038/s41598-026-47887-8.

## Introduction

Synthetic dyes are among the most persistent industrial pollutants due to their complex aromatic structures and high water solubility, which lead to widespread environmental and health issues. Industries such as textile manufacturing, leather processing, and printing release effluents containing synthetic dyes, many of which resist biological degradation and conventional wastewater treatment. Acid Blue 113 (AB113), an anionic sulfonated azo dye commonly used in textile dyeing, exemplifies this issue by exhibiting intense color even at low concentrations, potential toxicity, and resistance to biodegradation, all of which hinder light penetration and harm aquatic ecosystems in receiving waters^1^.

Traditional treatments such as coagulation–flocculation, advanced oxidation, and membrane filtration work well under certain conditions but often entail high operational costs, use large amounts of chemicals, and produce secondary waste streams. This has led to growing interest in adsorption as a practical, scalable remediation technique^2^. Adsorption-based processes offer flexibility, high removal efficiency, and the potential for adsorbent regeneration, making them appealing for dye cleanup in both batch and continuous systems^3^. However, the success of adsorption mainly depends on the adsorbent’s characteristics, including surface chemistry, porosity, and its interactions with dye molecules^4^.

Activated carbons and low-cost biosorbents, such as agricultural wastes, have been extensively studied for the removal of AB113. For instance, research using natural sorbents and activated carbon derivatives has shown varied adsorption capacities that depend on conditions such as pH, contact time, and temperature^5,6^. While these materials can achieve high equilibrium capacities, challenges persist in separating fine particles from treated water, pressure drops in packed systems, and performance decline after regeneration^7^. Recent studies have also explored advanced adsorbents such as porous carbons, functionalized polymers, and hybrid composites for dye and organic pollutant removal, demonstrating that surface functionalization and hierarchical porosity can significantly improve adsorption efficiency and selectivity (e.g., porous carbon materials, polymeric adsorbents, and hybrid composites)^8–11^. These systems highlight the importance of integrating tailored surface chemistry with accessible pore structures to enhance pollutant uptake in aqueous environments.

Emerging polymeric adsorbents offer a promising alternative, with tunable surface functionalities and customized porosity, thereby enhancing both adsorption efficiency and ease of processing^12^. Among these, cryogels, macroporous polymer networks created through cryopolymerization, feature interconnected pores that facilitate convective mass transport, rapid adsorption rates, low hydraulic resistance, and enable adsorption in continuous flow systems^13^. Functionalization of cryogels with reactive comonomers such as glycidyl methacrylate (GMA) allows for post-synthetic attachment of specific ligands, boosting selectivity and binding strength toward targeted pollutants^14^.

A widely studied cryogel platform is based on Poly(2-Hydroxyethyl methacrylate) (PolyHEMA) copolymerized with GMA. PolyHEMA provides a hydrophilic backbone, while GMA introduces epoxide groups that serve as versatile sites for covalent attachment of functional ligands under mild conditions^15^. This structural design allows precise control over surface functionality, supporting adsorption mechanisms that go beyond nonspecific interactions to include electrostatic and coordination binding^16^. Functional ligands capable of intense, selective interactions with anionic dyes are essential for effective adsorption. Poly(L-lysine) (PLL), a cationic polypeptide rich in primary amines, offers a high density of protonatable sites that can attract negatively charged sulfonate groups on AB113 through electrostatic interactions. Additionally, hydrogen bonding and secondary interactions can enhance binding strength, especially when paired with porous architectures that facilitate rapid mass transfer^17^. Although Poly(HEMA-GMA) cryogels and their amine-functionalized derivatives, such as Poly(HEMA-GMA)-PLL, have been previously investigated in adsorption and affinity systems, these studies primarily relied on electrostatic interactions as the dominant binding mechanism. Consequently, adsorption performance may decrease in complex aqueous matrices containing competing ions or high ionic strength. Therefore, introducing additional interaction mechanisms beyond electrostatic attraction is an important strategy to improve the adsorption stability and efficiency of persistent sulfonated azo dyes^18^.

Incorporating transition metal coordination sites into polymer matrices introduces complementary binding mechanisms. Transition metals such as Cu(II) can form stable chelates with ligand donors such as amines or heteroatoms on dye molecules, thereby enhancing selectivity and affinity, especially when electrostatic interactions are reduced by high ionic strength or competing ions in actual wastewater^19^. Recent reports on metal-functionalized porous materials and polymeric adsorbents have shown that coordination interactions can significantly enhance pollutant capture through multi-interaction adsorption mechanisms^20,21^. However, the integration of such coordination-active sites within macroporous cryogel architectures designed for dye adsorption remains relatively limited. Although metal-chelated adsorbents have been used for metal ion capture and biomolecule affinity separations, their integration into dye-adsorptive cryogel matrices is still underdeveloped^22^. While various polymeric and hybrid adsorbents have been explored for dye removal, few studies have combined macroporous cryogel architectures with both high-density cationic ligands and coordinated metal sites for improved anionic dye capture. This constitutes a significant gap, particularly for persistent sulfonated azo dyes such as AB113, where multiple interaction modes (electrostatic, hydrogen bonding, and coordination) can act synergistically to enhance adsorption performance^23^.

Here, we introduce a Poly(HEMA-GMA) cryogel functionalized sequentially with PLL and Cu(II), creating a novel adsorbent, Poly(HEMA-GMA)-PLL-Cu(II), for the removal of AB113. The macroporous cryogel scaffold enhances convective transport and minimizes diffusional limitations, while PLL provides a dense cationic environment that attracts anionic dye species. The chelation of Cu(II) ions onto PLL-anchored sites is expected to form coordination-binding centers that interact with the electron-donating groups of the azo dye, thereby boosting dye capture across diverse water chemistries^24^. Compared with previously reported Poly(HEMA-GMA) or Poly(HEMA-GMA)-PLL cryogel systems, the present approach integrates macroporous cryogel architecture, high-density cationic ligand functionality, and Cu(II)-based coordination binding within a single adsorbent platform. This multimodal interaction design is expected to improve adsorption stability under variable ionic strength conditions and enhance dye-capture efficiency beyond conventional electrostatic adsorption systems. This multi-modal interaction framework is hypothesized to lead to faster adsorption, higher equilibrium capacities, and improved regenerability compared to current adsorbents. This approach represents a significant innovation by integrating macroporosity, cationic ligand density, and metal coordination chemistry into a single, regenerable cryogel platform optimized for the removal of anionic dyes from water. In addition to high adsorption efficiency, adsorbents intended for wastewater treatment should ideally resist microbial colonization, which often leads to biofouling, pore blockage, and performance deterioration during prolonged operation. In this context, polymeric cryogels incorporating cationic ligands and metal-coordination sites may offer intrinsic antimicrobial properties, providing an added functional advantage for sustainable water treatment and long-term industrial effluent polishing applications^25^.

## Materials and methods

### Chemicals

2-Hydroxyethyl methacrylate (HEMA, 98%), glycidyl methacrylate (GMA, ≥ 99%), ethylene glycol dimethacrylate (EGDMA, 98%), sodium dodecyl sulfate (SDS, ≥ 99%), N, N,N′,N′-tetramethylethylenediamine (TEMED, ≥ 99.5%), ammonium persulfate (APS, ≥ 99.99%), Poly(L-lysine) hydrobromide (PLL, M_w_: 30.000–70.000, powder), copper(II) nitrate trihydrate [Cu(NO_3_)_2_·3 H_2_O], sodium chloride (NaCl, ≥ 99.0%), ethanol (≥ 99.8%), hydrochloric acid (37%, ACS reagent), sodium hydroxide (NaOH, ≥ 98%), and Acid Blue 113 (AB113) dye (dye content 50%) were purchased from Sigma-Aldrich (Steinheim, Germany). All chemicals were of analytical grade and used without further purification. Ultra-pure water (18.2 MΩ·cm) was used throughout all experiments.

### Synthesis of poly(HEMA-GMA) cryogels

Poly(HEMA-GMA) cryogels were synthesized by free-radical cryopolymerization at sub-zero temperatures. For the monomer phase, HEMA (5 mL), GMA (0.5 mL), and distilled water (6.5 mL) were mixed thoroughly until a homogeneous solution was obtained. Separately, the disperse phase was prepared by dissolving SDS (1.0 g) in distilled water (25.6 mL), followed by the addition of EGDMA (2.4 mL) as the crosslinking agent.

Both phases were combined and stirred to form a uniform precursor mixture, which was then equilibrated in an ice bath for 10–15 min. Polymerization was initiated by adding APS (20 mg) and TEMED (100 µL). The reaction mixture was transferred into molds and allowed to polymerize at − 20 °C for 24 h.

After polymerization, the cryogels were thawed at room temperature, cut into disc-shaped membranes, and extensively washed with distilled water using a rotator (Multi Bio RS-24, Biosan, Riga, Latvia) at 10 rpm to remove SDS and residual unreacted species^26^.

### PLL grafting and Cu(II) functionalization of poly(HEMA-GMA) cryogels

**Fig. 1:**
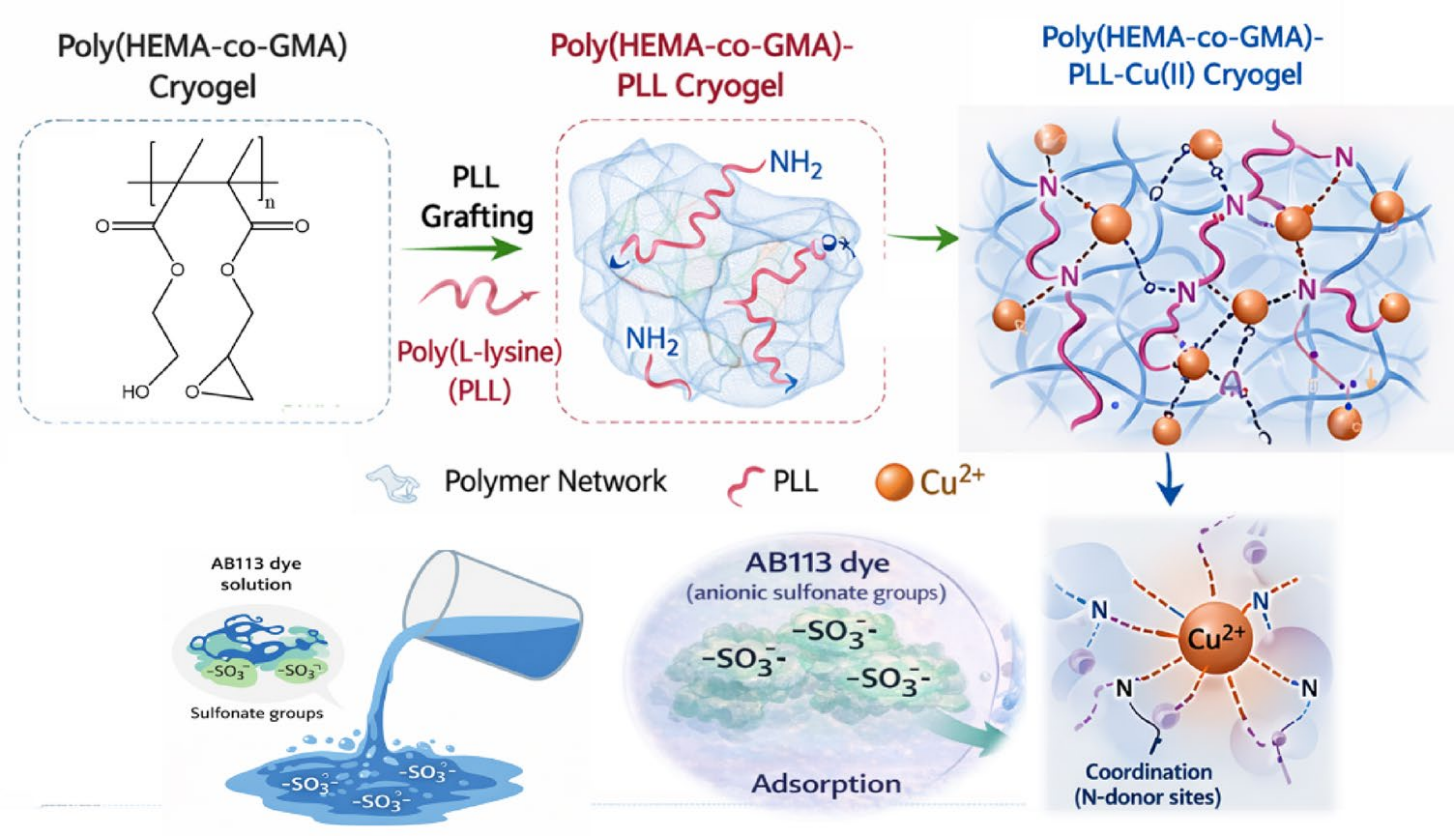
Schematic representation of the preparation and adsorption mechanism of the Poly(HEMA-co-GMA)-PLL-Cu(II) cryogel. PLL is grafted onto the Poly(HEMA-co-GMA) cryogel to introduce amine (–NH_2_) groups, which subsequently coordinate with Cu(II) ions to form the functionalized cryogel network. During adsorption, AB113 dye molecules, containing anionic sulfonate (–SO_3_^−^) groups, interact with the cryogel matrix through electrostatic attraction with protonated amines and possible coordination interactions involving Cu(II) centers.

Before Cu(II) loading, Poly(HEMA-GMA) cryogels were chemically activated to enable efficient PLL grafting (Fig. 1). Briefly, 20 cryogel discs (approximately 0.28 g) were treated with 1 M NaOH (10 mL) under gentle stirring for 2 h to open the epoxy rings of GMA units. The activated cryogels were thoroughly washed with distilled water until the pH reached neutrality^27,28^.

The cryogels were then immersed in a PLL solution (25 mg mL^−1^, 10 mL) and incubated for 24 h at room temperature, allowing PLL chains to graft covalently onto the cryogel matrix via epoxy ring-opening reactions. After PLL modification, the cryogels were transferred into an aqueous Cu(NO_3_)_2_·3 H_2_O solution (5 mg mL^−1^, 10 mL) and gently stirred for 6 h to facilitate Cu(II) coordination via PLL amine groups. The development of an intense blue coloration visually confirmed the successful immobilization of Cu(II). The resulting Poly(HEMA-GMA)-PLL-Cu(II) cryogels were washed repeatedly with distilled water and finally rinsed with ethanol to remove loosely bound metal ions. All samples were stored at 4 °C before adsorption experiments.

###  Characterization studies

A comprehensive set of physicochemical characterization techniques was employed to evaluate structural, chemical, and morphological changes occurring in Poly(HEMA-GMA) cryogels following PLL grafting and Cu(II) functionalization. These analyses were conducted to elucidate how surface modification and metal coordination influence cryogel properties relevant to the adsorption of AB113 dye from aqueous media.

Swelling behavior and water retention capacity (*WRC*) were determined to assess network porosity and hydrophilicity. *WRC* values were calculated according to:

1 $$\:WRC\:\left(\%\right)\:=\:({W}_{s}\:\--\:{W}_{d})\:/\:{W}_{s}\:\times\:\:100$$

*W*_*s*_: the weight of the swollen cryogel at equilibrium, *W*_*d*_: the weight after drying or centrifugation.

Fourier-transform infrared (FT-IR) spectroscopy was used to confirm successful copolymerization, epoxy activation, PLL grafting, and Cu(II) coordination. FT-IR spectra were recorded in the range of 4000–400 cm^−1^ using a Thermo Scientific Nicolet 6700 spectrometer (ATR mode, 4 cm^−1^ resolution).

The macroporous morphology and interconnected pore structure of the cryogels were examined by scanning electron microscopy (SEM). Samples were freeze-dried, gold-sputtered, and imaged using a FEI Quanta 450 FEG microscope at an accelerating voltage of 10–20 kV.

Thermogravimetric analysis (TGA) was carried out using a Shimadzu DTG-60 H instrument under nitrogen atmosphere from 30 °C to 700 °C at a heating rate of 10 °C min^−1^ to evaluate thermal stability and compositional changes induced by PLL and Cu(II) incorporation.

Brunauer–Emmett–Teller (BET) analysis was performed to determine specific surface area and pore characteristics using a Quantachrome Autosorb^®^ iQ-Chemi analyzer. Before measurements, samples were degassed at 90 °C for 12 h.

X-ray photoelectron spectroscopy (XPS) analysis was performed to investigate the surface elemental composition and chemical states of the cryogel structures after PLL grafting and Cu(II) coordination. The spectra were recorded using a PHI 5000 VersaProbe XPS system.

The amount of grafted PLL was quantified via elemental nitrogen analysis using an Elementar Vario PYRO Cube analyzer, while Cu(II) loading capacity was determined by inductively coupled plasma optical emission spectroscopy (ICP-OES; Spectro Arcos, Kleve, Germany).

### Determination of antibacterial and antifungal activity

The antibacterial and antifungal activities of the synthesized cryogels were evaluated by determining their minimum inhibitory concentrations (MICs) using the broth microdilution method in 96-well microplates, following standard protocols described by Schwalbe et al.^29^ and the National Committee for Clinical Laboratory Standards^30^.

Gram-positive bacteria *Bacillus subtilis* ATCC 6633, *Brevibacillus brevis* ATCC 35,690, and *Listeria monocytogenes* NCTC 5348; Gram-negative bacteria *Escherichia coli* ATCC 25,922, Enterobacter *aerogenes* CCU 2531, and *Salmonella typhimurium* NRRLE 4413; and the fungal strains *Candida glabrata* ATCC 15,126 and *Candida tropicalis* ATCC 13,803 were used as test microorganisms. All strains were stored at − 80 °C in stock tubes containing 25% (v/v) glycerol until use.

Cryogel extracts were prepared by dispersing the synthesized cryogels in distilled water and heating to 60 °C to facilitate the release of active components into the aqueous phase. For microbial activation, bacterial strains were cultured on Mueller–Hinton Agar (MHA), while fungal strains were grown on Sabouraud Dextrose Agar (SDA) (Difco). Prior to MIC testing, bacterial cultures were transferred into Mueller–Hinton Broth (MHB) and fungal cultures into Sabouraud Dextrose Broth (SDB) (Difco), and incubated overnight at 37 °C for bacteria and 28 °C for fungi. The turbidity of each microbial suspension was adjusted to 0.5 McFarland standard, corresponding to approximately 1.5 × 10⁸ CFU mL^−1^.

For MIC determination, 100 µL of the standardized microbial suspension and 100 µL of the cryogel extract at varying concentrations were added to each well of the microplate. Wells containing only microbial suspension and distilled water served as growth and negative controls, respectively. After incubation, MIC values were recorded as the lowest concentration of cryogel extract (µg mL^−1^) that showed no visible microbial growth. All experiments were performed in duplicate. Amoxicillin and tetracycline were used as reference antibiotics for antibacterial activity.

### Adsorption of AB113

Adsorption experiments were conducted to evaluate the removal efficiency of Poly(HEMA-GMA)-PLL-Cu(II) cryogels for AB113 from aqueous solutions under batch conditions. A stock solution of AB113 (1000 mg L^−1^) was prepared in ultrapure water, and working solutions with the desired initial concentrations were prepared by appropriate dilution. The initial dye concentration was determined by UV–Vis spectrophotometry at AB113’s maximum absorbance (565 nm).

In a typical experiment, one cryogel disc (average dry mass: 14 ± 0.4 mg) was immersed in 10 mL of AB113 solution, and the effects of solution pH, contact time, initial dye concentration, and temperature on adsorption behavior were systematically examined. The pH of the dye solutions was adjusted with 0.1 M HCl or 0.1 M NaOH before adsorption. Adsorption experiments were performed at controlled temperatures using a thermostated orbital shaker, with the system agitated at a constant shaking speed to ensure uniform mass transfer.

At predetermined contact times, cryogel discs were removed from the solutions, and the residual AB113 concentration was measured spectrophotometrically. The decrease in absorbance compared to the initial value indicated successful adsorption of AB113 onto the cryogel matrix. All adsorption experiments were conducted in triplicate, and the reported values reflect the average of three independent measurements. The adsorption capacity of the cryogels toward AB113 was calculated using the following Eq. 

2 $$\:q\:=\:\left(\right(C_{0}\:\--\:C_{\mathrm{t}})\:\times\:\:V)\:/\:m$$

Where *C₀* is the initial AB113 concentration (mg L^−1^), *C*_*t*_ is the dye concentration (mg L^−1^) at time *t*, *V* is the volume of the dye solution (L), and *m* is the dry mass of the cryogel (g). This equation quantifies the amount of AB113 (mg g^−1^) removed from the water and adsorbed onto the cryogel.

###  Desorption and regeneration experiments

Desorption and regeneration studies were conducted to assess the reusability of Poly(HEMA-GMA)-PLL-Cu(II) cryogels after AB113 adsorption. After each adsorption cycle, the dye-loaded cryogel discs were carefully rinsed with ultrapure water to remove physically trapped or loosely bound dye molecules. Desorption was then performed by immersing the cryogels in 10 mL of a mixed desorbing solution containing 0.5 M NaCl and 30% (v/v) ethanol, with the pH adjusted to 10.0 using 0.01 M NaOH. The system was agitated at 200 rpm and 25 °C for 60 min to disrupt electrostatic interactions, weaken hydrogen bonds, and suppress hydrophobic contributions responsible for AB113 binding^31^. After desorption, the cryogels were separated from the solution, and the concentration of desorbed AB113 was measured using UV–Vis spectrophotometry at its characteristic maximum absorption wavelength. The regenerated cryogels were then rinsed with ultrapure water until the pH reached neutrality and reused in the next adsorption cycle under identical conditions. Desorption efficiency (%) was calculated as the ratio of the amount of dye released during desorption to the initial amount adsorbed onto the cryogel.

###  Adsorption isotherms/kinetics and thermodynamic analysis

Equilibrium adsorption experiments were conducted at 10, 30, and 50 °C, and the equilibrium data were fitted to the Langmuir, Freundlich, Temkin, and Dubinin–Radushkevich (D–R) isotherm models. Langmuir assumes monolayer adsorption on a homogeneous surface; Freundlich describes heterogeneous adsorption; Temkin accounts for adsorbate–adsorbent interactions and a coverage-dependent heat of adsorption; D–R enables estimation of mean adsorption energy to infer whether adsorption is predominantly physical or involves stronger specific interactions. Kinetic data were analyzed using pseudo-first-order (PFO) and pseudo-second-order (PSO) models to determine the primary rate-controlling mechanism. A better PSO fit generally suggests that adsorption is mainly controlled by active-site availability and surface interactions, rather than by external mass transfer alone.

 Thermodynamic parameters, including Gibbs free energy change (ΔG°), enthalpy change (ΔH°), and entropy change (ΔS°), were determined from temperature-dependent equilibrium data using the Van’t Hoff approach. These parameters were used to assess spontaneity, heat effects, and changes in disorder during adsorption, respectively. All the equations and parameters necessary for these models and calculations were presented in the supplementary file (Table S1 and Table S2).

## Results and discussions

### Characterization results

**Fig. 2:**
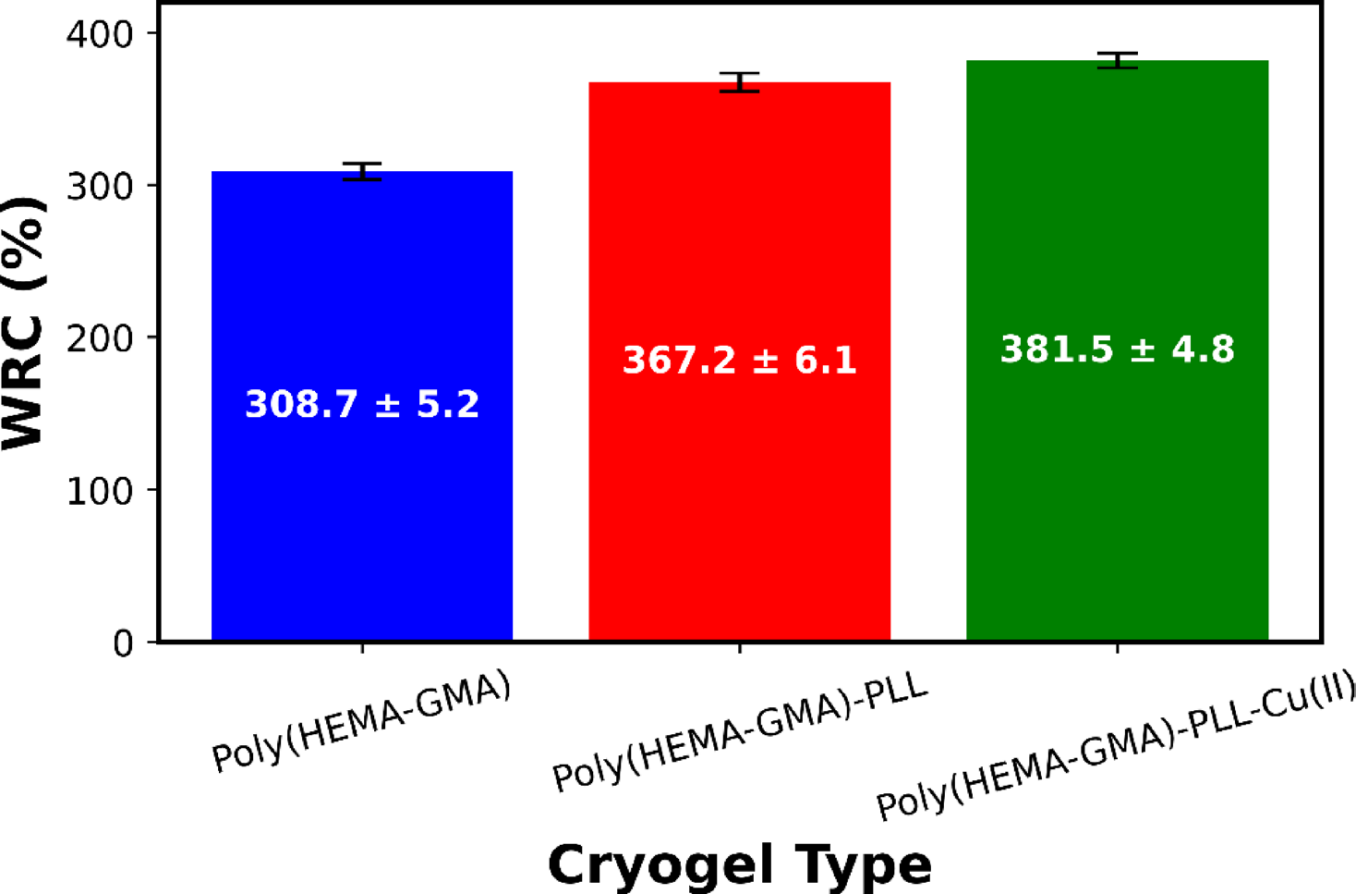
WRC (%) of cryogels with different surface functionalities: pristine Poly(HEMA-GMA), Poly(HEMA-GMA)-PLL, and Poly(HEMA-GMA)-PLL-Cu(II). Bars represent mean values and error bars indicate standard deviation (*n* = 3).

The *WRC* results indicate an apparent increase in hydrophilicity and swelling behavior with successive chemical modifications to the cryogel structure (Fig. 2). Pristine Poly(HEMA-GMA) displays a high *WRC* value of 308.7%, which is typical for macroporous cryogels containing hydrophilic hydroxyl groups and an interconnected pore network capable of holding large amounts of water.

After modification with PLL, the *WRC* significantly increases to 367.2% for Poly(HEMA-GMA)-PLL. This rise is mainly due to the presence of numerous amino groups along the PLL chains, which strengthen hydrogen bonding with water molecules and boost osmotic swelling within the cryogel matrix. Additionally, the flexible PLL chains enhance water uptake while maintaining the macroporous structure.

The highest *WRC* value (381.5%) is observed for Poly(HEMA-GMA)-PLL-Cu(II), indicating that Cu(II) coordination further improves water retention. Coordination between Cu(II) ions and PLL amino groups likely causes local structural changes and the formation of additional hydration shells, enabling more water to be immobilized within the cryogel network. The lack of a decrease in *WRC* suggests that metal chelation does not cause excessive crosslinking or pore collapse.

The consistent increase in *WRC* with PLL functionalization and subsequent Cu(II) chelation confirms the successful modification of the cryogels. It emphasizes their strong affinity for water, which is advantageous for adsorption- and mass-transfer-controlled processes in aqueous environments.

**Fig. 3:**
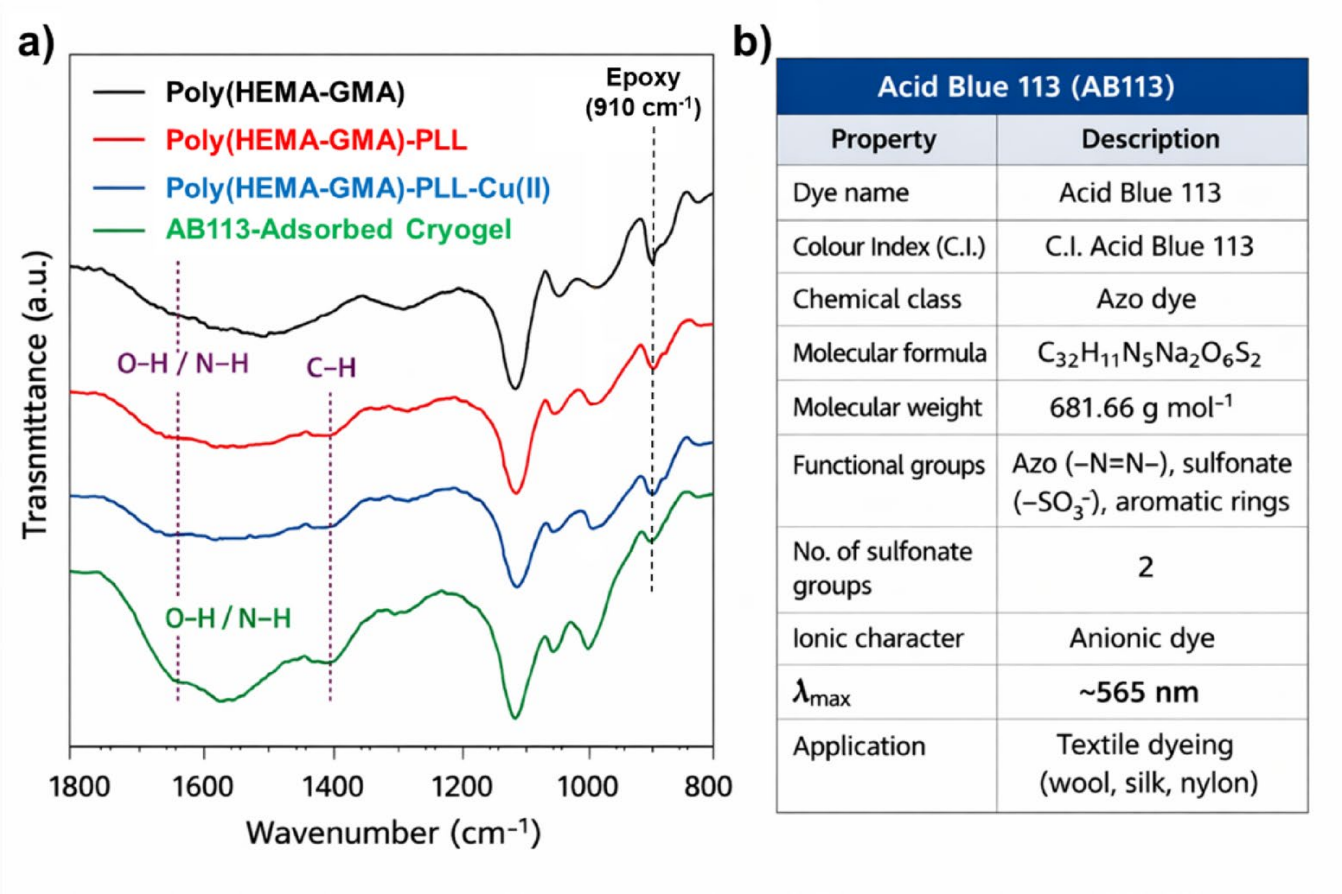
FT-IR spectra illustrating the stepwise functionalization of the cryogel and the interaction of AB113 dye with the modified adsorbent. (**a**) Stacked FT-IR spectra of Poly(HEMA-GMA), Poly(HEMA-GMA)-PLL, Poly(HEMA-GMA)-PLL-Cu(II), and AB113-adsorbed Poly(HEMA-GMA)-PLL-Cu(II) cryogels. (**b**) Physicochemical characteristics of the Acid Blue 113 dye used as the model pollutant in the adsorption experiments.

Figure 3a presents the stacked FT-IR transmittance spectra of Poly(HEMA-GMA), Poly(HEMA-GMA)-PLL, Poly(HEMA-GMA)-PLL-Cu(II), and AB113-adsorbed Poly(HEMA-GMA)-PLL-Cu(II) cryogels, clearly illustrating the progressive chemical modifications and dye interaction within the cryogel system. The spectrum of Poly(HEMA-GMA) exhibits the characteristic ester carbonyl stretching band at ~ 1725 cm^−1^, along with C–O–C stretching vibrations in the 1260–1070 cm^−1^ region, confirming the successful formation of the HEMA-GMA polymeric backbone. The broad absorption around 3400 cm^−1^ is attributed to overlapping O–H stretching vibrations of the hydrophilic cryogel matrix.

Following PLL grafting, the Poly(HEMA-GMA)-PLL cryogel exhibits distinct amide bands, with amide I and amide II vibrations appearing around 1650 and 1540 cm^−1^, respectively. These bands clearly indicate successful immobilization of PLL on the cryogel surface. Additionally, the increased intensity and broadening in the 3300–3400 cm^−1^ range suggest N–H stretching vibrations from PLL chains, further confirming the presence of amine-rich functionalities. Although the epoxy vibration band of GMA (~ 910 cm^−1^) remains observable after PLL grafting, this does not contradict successful modification. The grafting reaction primarily occurs at accessible epoxy sites on the surface, while a fraction of epoxide groups remain unreacted within the cryogel network, resulting in only partial changes in intensity rather than complete disappearance of the epoxy band^32^.

Upon Cu(II) loading, clear spectral changes appear in the Poly(HEMA-GMA)-PLL-Cu(II) spectrum. The amide I and II bands show slight shifts and changes in intensity, indicating coordination interactions between Cu(II) ions and the amide/amine groups of PLL. Additionally, new absorption features below approximately 650 cm^−1^ emerge, attributed to Cu–N and Cu–O vibrations, providing evidence of metal–ligand complex formation within the cryogel network^33^. These interactions suggest that Cu(II) ions are effectively chelated by PLL functionalities rather than simply being physically adsorbed.

After adsorption of AB113, the FT-IR spectrum of the dye-loaded cryogel exhibits additional spectral features associated with the dye molecules (Fig. 3a, b). In particular, bands attributed to aromatic C = C vibrations (~ 1600 cm^−1^) and azo (–N = N–) stretching vibrations (~ 1490–1500 cm^−1^) become more pronounced. Furthermore, characteristic sulfonate (SO_3_^−^) stretching vibrations appear in the 1030–1120 cm^−1^ region^34^. Slight shifts in the N–H and S = O bands suggest electrostatic interactions between the sulfonate groups of AB113 and the protonated amine groups of PLL, while possible coordination interactions with Cu(II) sites may also contribute to the adsorption process.

Collectively, the FT-IR results show a stepwise structural change from the base Poly(HEMA-GMA) cryogel to the PLL-functionalized and Cu(II)-chelated forms, and further confirm the successful interaction between AB113 dye molecules and the functionalized cryogel matrix through electrostatic and coordination-based interactions.

**Fig. 4:**
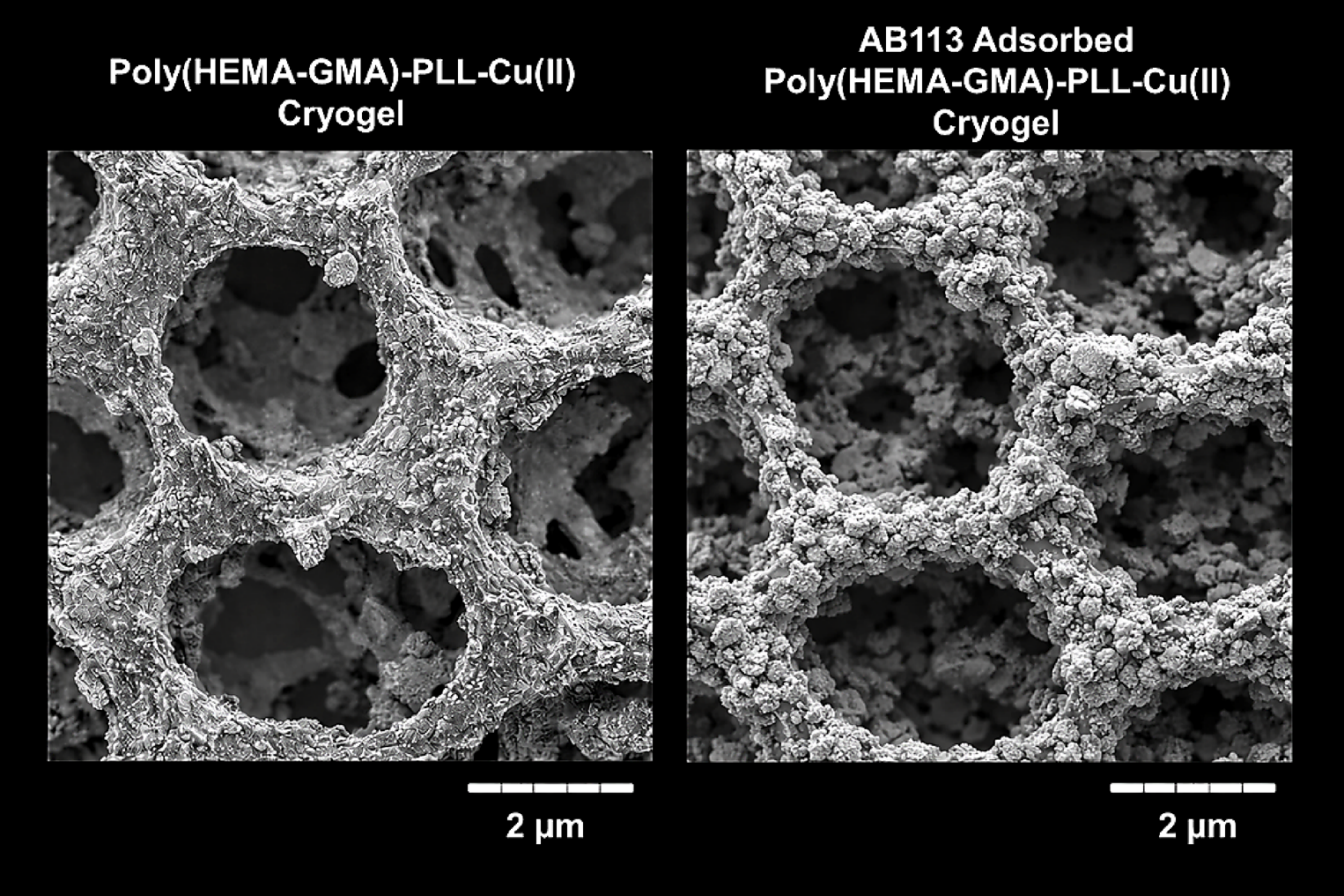
SEM images of Poly(HEMA-GMA)-PLL-Cu(II) cryogels illustrating the morphological changes before and after adsorption: (**a**) pristine cryogel prior to adsorption, exhibiting a highly interconnected macroporous structure with open channels that facilitate mass transfer; (**b**) cryogel after AB113 adsorption, where partial pore coverage and surface deposition of dye molecules are observed, indicating successful interaction between the dye and functionalized cryogel matrix. The slight reduction in pore openness and increased surface roughness after adsorption suggest effective binding of AB113 through electrostatic interactions and Cu(II)-mediated coordination.

Figure 4 presents SEM images of the pristine Poly(HEMA-GMA)-PLL-Cu(II) cryogel and the AB113-adsorbed Poly(HEMA-GMA)-PLL-Cu(II) cryogel. The unadsorbed Poly(HEMA-GMA)-PLL-Cu(II) cryogel exhibits a well-defined, highly interconnected macroporous structure with relatively smooth pore walls and open pore cavities. This morphology is characteristic of cryogels formed under cryopolymerization conditions and is favorable for rapid mass transfer, low diffusion resistance, and effective interaction between the adsorbent surface and solute molecules.

After AB113 adsorption, noticeable morphological changes are observed. The macroporous framework remains intact, indicating that the cryogel retains its structural stability during adsorption. However, the pore walls and internal surfaces appear significantly roughened and partially covered with irregular aggregates attributed to adsorbed AB113 molecules. These deposits are distributed along pore edges and within pore interiors, suggesting that dye uptake occurs not only on the external surface but also throughout the three-dimensional cryogel network.

The increased surface roughness and partial pore filling after adsorption provide clear visual evidence of successful AB113 immobilization. At the same time, the persistence of open pores indicates that adsorption does not cause severe pore blockage or structural collapse. This combination of preserved macroporosity and extensive surface coverage supports the suitability of the Poly(HEMA-GMA)-PLL-Cu(II) cryogel for efficient dye adsorption under aqueous conditions.

**Fig. 5:**
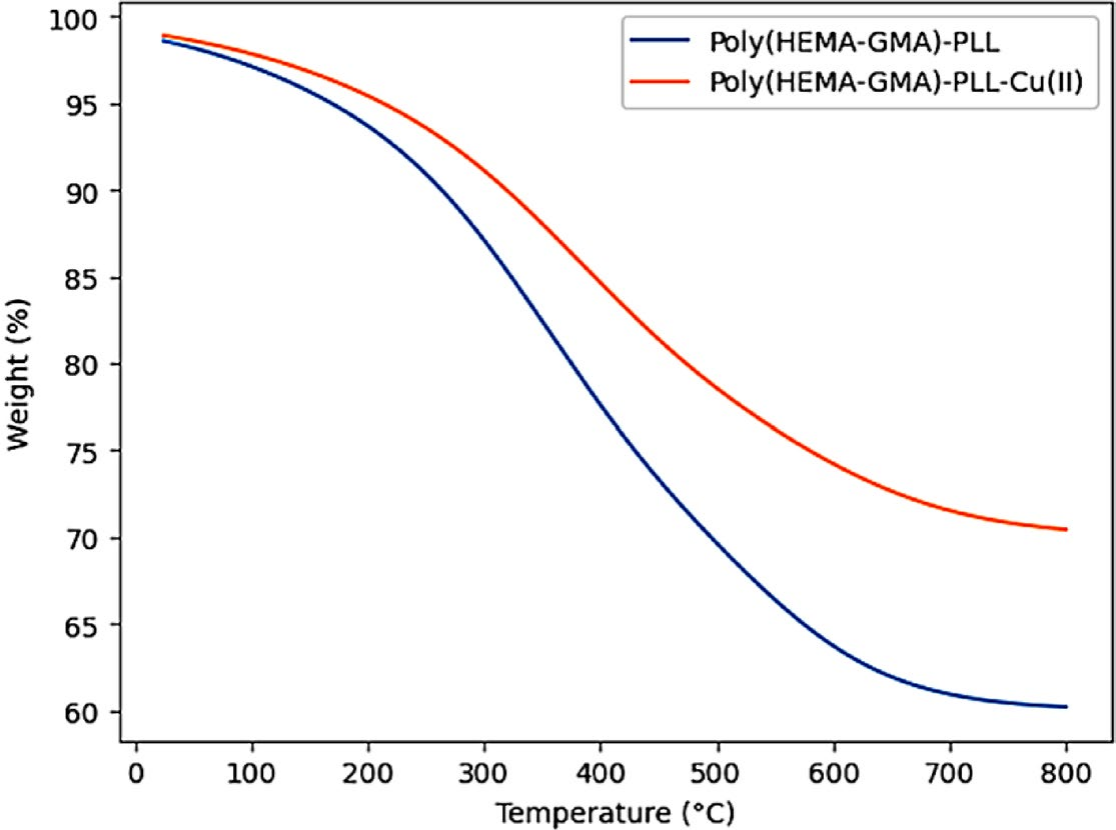
TGA curves of Poly(HEMA-GMA)-PLL and Poly(HEMA-GMA)-PLL-Cu(II) cryogels recorded under a nitrogen atmosphere, showing their thermal degradation behavior.

Figure 5 shows the comparative TGA profiles of Poly(HEMA-GMA)-PLL and Poly(HEMA-GMA)-PLL-Cu(II) cryogels recorded over the temperature range of 25–800 °C. Both samples display a typical multi-step thermal degradation behavior common to polymer-based cryogels.

The initial minor weight loss observed below about 150 °C for both cryogels is due to the removal of physically adsorbed and weakly bound water within the macroporous structure. This step is less pronounced in the Cu(II)-chelated cryogel, indicating stronger water–polymer interactions due to metal coordination.

The primary degradation stage occurs between roughly 250 and 450 °C and involves the thermal breakdown of the polymer backbone and PLL side chains. In this range, Poly(HEMA-GMA)-PLL shows a sharper mass loss, while Poly(HEMA-GMA)-PLL-Cu(II) displays a delayed and milder degradation pattern. This shift indicates the stabilizing influence of Cu(II) coordination, which limits polymer chain mobility and boosts resistance to thermal scission.

At temperatures above 500 °C, Poly(HEMA-GMA)-PLL-Cu(II) maintains a noticeably higher residual mass compared to the non-chelated cryogel. This higher char yield is linked to the presence of inorganic Cu-containing species and the formation of more thermally stable crosslinked regions. The increased residue confirms that the addition of Cu(II) enhances the cryogel’s thermal stability without causing premature degradation^35^.

These results show that Cu(II) chelation greatly improves the thermal stability of Poly(HEMA-GMA)-PLL cryogels, confirming their suitability for applications requiring durability at high temperatures or repeated regeneration.

**Fig. 6:**
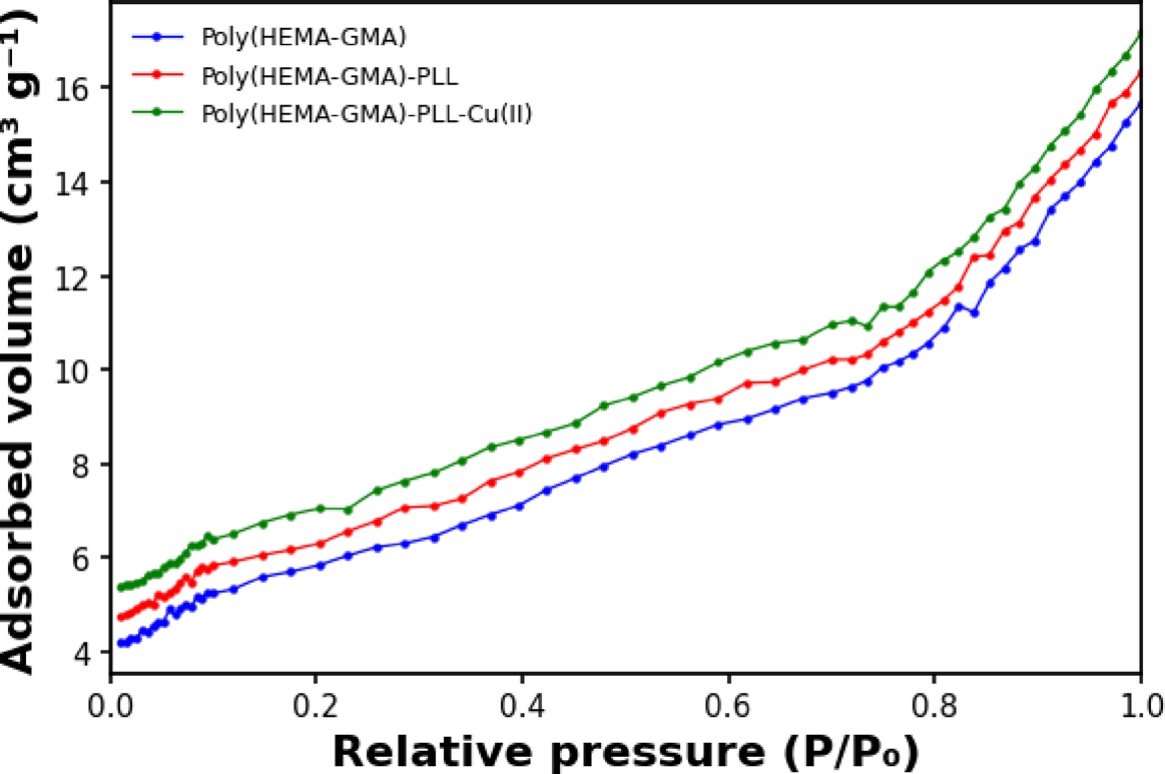
Nitrogen adsorption isotherms of Poly(HEMA-GMA), Poly(HEMA-GMA)-PLL, and Poly(HEMA-GMA)-PLL-Cu(II) cryogels obtained from BET surface area analysis. Measurements were performed using N_2_ adsorption at 77 K over a relative pressure (P/P₀) range of 0.01–1.0.

The nitrogen adsorption isotherms presented in Fig. 6 indicate a progressive increase in adsorbed volume following each modification step of the cryogel structure. Pristine Poly(HEMA-GMA) exhibits a BET surface area of 6.45 m^2^ g^−1^, which is typical for macroporous cryogels where the pore architecture is dominated by large interconnected channels rather than micro- or mesoporous domains. In such systems, relatively low BET surface areas are commonly observed despite their high swelling capacity and water permeability^36,37^.

After PLL modification, the surface area increased to 7.12 m^2^ g^−1^ for Poly(HEMA-GMA)-PLL. The increase can be attributed to the attachment of PLL chains to the cryogel backbone, which likely increases surface roughness and generates additional accessible adsorption sites on the pore walls without blocking the macroporous structure.

 The highest surface area (7.85 m^2^ g^−1^) was obtained for Poly(HEMA-GMA)-PLL-Cu(II). Coordination of Cu(II) with the amino groups of PLL may induce subtle structural rearrangements within the polymer network, suppress polymer chain aggregation, and stabilize a more open structure. In addition, metal–ligand interactions may increase pore-wall rigidity, allowing the cryogel to better preserve its surface accessibility during drying and BET measurements. The increase in surface area after Cu(II) chelation indicates that metal incorporation does not cause pore collapse but instead improves the structural definition of the cryogel surface, thereby enhancing adsorption by providing more accessible active sites. Quantitative pore structure parameters such as pore volume and average pore diameter derived from BET analysis are presented in the supplementary file (Table S3).

**Fig. 7:**
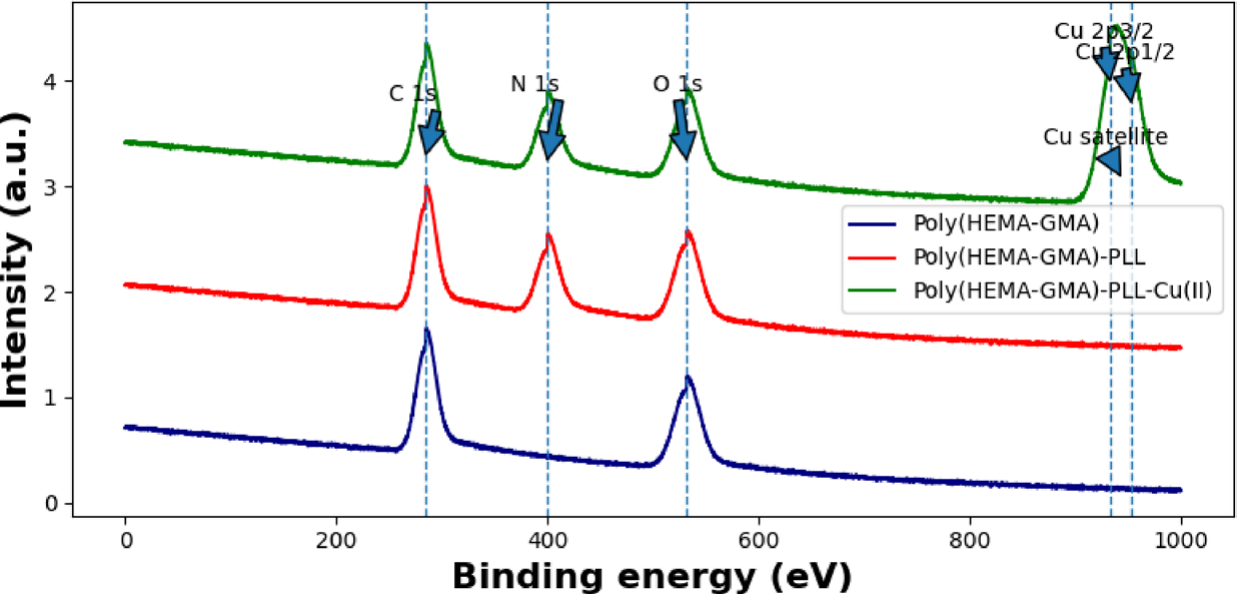
XPS survey spectra of Poly(HEMA-GMA), Poly(HEMA-GMA)-PLL, and Poly(HEMA-GMA)-PLL-Cu(II) cryogels, demonstrating the progressive surface modification after PLL grafting and Cu(II) coordination.

The XPS survey spectra shown in Fig. 7 reveal the progressive surface modification from Poly(HEMA-GMA) to Poly(HEMA-GMA)-PLL and finally to the Poly(HEMA-GMA)-PLL-Cu(II) complex. In the spectrum of pristine Poly(HEMA-GMA), the dominant signals at approximately 285 eV and 532 eV correspond to the C 1 s and O 1 s peaks originating from the polymer backbone containing ester and hydroxyl groups. After the introduction of PLL, a new N 1 s peak appears around 399–401 eV, indicating the successful incorporation of amine groups onto the polymer surface. In the Poly(HEMA-GMA)-PLL-Cu(II) structure, additional peaks assigned to Cu 2p_3_/_2_ (~ 933–934 eV) and Cu 2p₁/_2_ (~ 953–954 eV), together with characteristic Cu satellite features, are clearly observed^38^. These signals confirm the presence of Cu(II) species coordinated with the nitrogen-containing PLL chains, supporting the successful formation of the Poly(HEMA-GMA)-PLL-Cu(II) complex.

An elemental nitrogen content of 8.5 wt% strongly indicates that the Poly(HEMA-GMA) scaffold was successfully enriched with amine-bearing PLL chains, resulting in a high density of cationic and ligating sites throughout the macroporous network. This level of nitrogen indicates extensive surface and pore-wall functionalization rather than just a minor surface coating, which is important because it means AB113 can access a large number of interaction sites under both aqueous flow and batch conditions.

Quantitatively, the PLL (ligand) amount of 0.389 mmol g^−1^ and Cu detected as Cu(II) at 0.274 mmol g^−1^ indicate a substantial metal loading on the PLL-modified cryogel. The Cu-to-polymer molar ratio is approximately 0.70 (0.274/0.389), suggesting that a significant portion of the available coordinating groups is engaged in copper binding, nearing saturation of the accessible chelation sites within the material. From a functional perspective, this high occupancy promotes the formation of numerous metal–ligand centers that can enhance electrostatic attraction (from protonated amines) with specific coordination-assisted interactions toward electron-donating parts of the dye, thereby improving uptake, especially under conditions where purely electrostatic binding may be weaker (e.g., high ionic strength).

Finally, the fact that copper is identified as Cu(I), even if the loading step uses Cu(II), can indicate a change in copper speciation after immobilization, such as partial reduction during binding or processing, or an analytical speciation assignment. Either way, it confirms that copper is not just physically trapped but present in a chemically bound form at high density. This combination of high nitrogen content and high copper loading supports a multi-modal adsorption surface (cationic and coordination-capable), likely improving the affinity and robustness of AB113 capture in realistic water matrices.

###  Adsorption/desorption studies

**Fig. 8:**
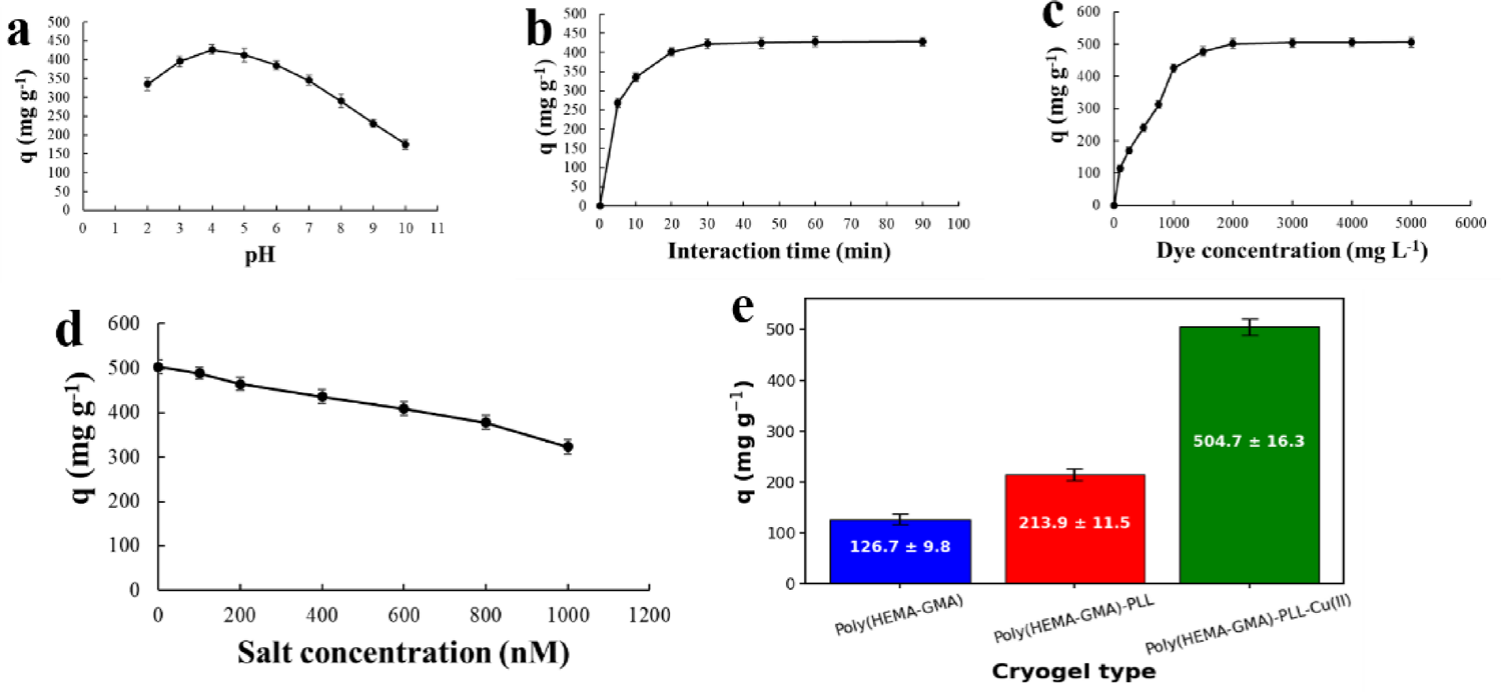
Adsorption performance of Poly(HEMA-GMA)-PLL-Cu(II) cryogels toward AB113: (**a**) effect of solution pH, (**b**) effect of contact time, (**c**) effect of initial dye concentration, (**d**) effect of ionic strength (salt concentration), and (**e**) comparison of adsorption capacities of different cryogel structures (Poly(HEMA-GMA), Poly(HEMA-GMA)-PLL, and Poly(HEMA-GMA)-PLL-Cu(II)). Adsorption experiments were performed using one cryogel disc (dry mass ≈ 14 mg) in 10 mL AB113 solution. All adsorption experiments were performed in triplicate (*n* = 3), and error bars represent the standard deviation of the measurements.

The effect of pH on the adsorption capacity of AB113 onto the Poly(HEMA-GMA)-PLL-Cu(II) cryogel was investigated at an initial dye concentration of 1000 mg L^−1^ and a contact time of 60 min. The adsorption capacity shows a pronounced dependence on solution pH, exhibiting a bell-shaped profile across the studied range (Fig. 8a). At acidic pH values, the adsorption capacity increases from 335.3 mg g^−1^ at pH 2.0 to a maximum of 426.6 mg g^−1^ at pH 4.0. This behavior is attributed to strong electrostatic attraction between the anionic sulfonate groups of AB113 and the positively charged amino groups of PLL, as well as to the presence of accessible Cu(II) coordination sites that enhance dye binding^39^.

A slight decrease in adsorption capacity is observed at pH 5.0 (412.5 mg g^−1^), indicating the beginning of reduced surface protonation. As the pH increases further, the adsorption capacity declines progressively, reaching 175.4 mg g^−1^ at pH 10.0. Under neutral and alkaline conditions, deprotonation of amino groups on the cryogel matrix diminishes the positive surface charge, thereby weakening electrostatic interactions with AB113. In addition, competition with hydroxide ions and possible alterations in the Cu(II) coordination environment contribute to the reduced adsorption efficiency at higher pH values. These results demonstrate that mildly acidic conditions provide the most favorable environment for AB113 adsorption onto Poly(HEMA-GMA)-PLL-Cu(II) cryogels.

Figure 8b illustrates the effect of interaction time on the adsorption of AB113 onto the Poly(HEMA-GMA)-PLL-Cu(II) cryogel at an initial dye concentration of 1000 mg L^−1^ and pH 4.0. The adsorption capacity increases rapidly during the initial stage, reaching 268.4 mg g^−1^ within the first 5 min, indicating a high affinity between AB113 molecules and the readily available active sites on the cryogel surface. This rapid uptake continues for up to 20 min, at which point the adsorption capacity rises sharply to 401.6 mg g^−1^, reflecting efficient mass transfer and strong interactions, such as electrostatic attraction and Cu(II)–dye coordination.

After 30 min, the adsorption capacity approaches 422.3 mg g^−1^, and further increases become marginal, demonstrating that most of the accessible binding sites are already occupied. Between 30 and 90 min, only slight changes are observed, with the adsorption capacity gradually increasing to 427.2 mg g^−1^ at 60 min and reaching 428.1 mg g^−1^ at 90 min, confirming the establishment of adsorption equilibrium. The minimal improvement in adsorption capacity after 30 min, along with overlapping error bars, indicates that 30 min is enough to reach near-equilibrium conditions for AB113 adsorption under the tested conditions. This behavior underscores the rapid kinetics and practical effectiveness of the Poly(HEMA-GMA)-PLL-Cu(II) cryogel, making it a promising adsorbent for rapid dye removal.

Figure 8c illustrates the effect of initial dye concentration on the adsorption capacity of the cryogel system at pH 4.0 with a fixed contact time of 30 min. As the dye concentration increases from 100 to 1000 mg L^−1^, the adsorption capacity rises sharply from 113.4 to 425.2 mg g^−1^, indicating efficient utilization of the available active sites on the cryogel surface. This rapid increase suggests a strong driving force for mass transfer at lower concentrations, where a high concentration gradient between the solution and the adsorbent promotes fast dye uptake.

Further increasing the concentration to 1500–2000 mg L^−1^ results in a more gradual increase in adsorption capacity, reaching 501.6 mg g^−1^, which reflects the progressive occupation of high-affinity binding sites. Beyond 2000 mg L^−1^, the adsorption capacity approaches a plateau, with only marginal changes observed up to 5000 mg L^−1^ (506.2 mg g^−1^). This behavior indicates near-saturation of the accessible adsorption sites within the cryogel matrix under the experimental conditions used.

The slight fluctuations in adsorption capacity values at higher concentrations lie within the experimental error and suggest that equilibrium is established within 30 min. The clear plateau region highlights the high adsorption efficiency and capacity of the cryogel, while also implying a finite number of energetically favorable binding sites. These results demonstrate that the system exhibits strong affinity toward the dye and achieves high loading even at relatively moderate concentrations, supporting its suitability for treating dye-rich aqueous solutions.

Figure 8d illustrates the effect of ionic strength on the adsorption capacity of AB113 onto the Poly(HEMA-GMA)-PLL-Cu(II) cryogel. The experiments were carried out at an initial dye concentration of 2000 mg L^−1^ with an interaction time of 60 min. As the salt concentration increases from 0 to 1000 nM, the adsorption capacity gradually decreases from 503.2 mg g^−1^ to 323.1 mg g^−1^.

The highest adsorption capacity is observed in the absence of salt, indicating that electrostatic interactions dominate the adsorption mechanism. The addition of salt introduces competing ions into the solution, which partially screen the electrostatic attraction between the negatively charged sulfonate groups of AB113 and the positively charged amino groups of the cryogel matrix. Consequently, the effective interaction between the dye molecules and the adsorption sites becomes weaker.

A progressive decrease in adsorption capacity is observed with increasing ionic strength, reaching 489.5 mg g^−1^ at 100 nM and 464.7 mg g^−1^ at 200 nM, then declining to 409.3 mg g^−1^ at 600 nM. At the highest salt concentration tested (1000 nM), the adsorption capacity decreases to 323.1 mg g^−1^, corresponding to an approximate 36% reduction compared with salt-free conditions. This behavior suggests that ionic strength partially suppresses electrostatic adsorption; however, the cryogel still retains a relatively high adsorption capacity even at elevated salt levels. Such performance indicates that, in addition to electrostatic attraction, other interactions such as Cu(II)–dye coordination and hydrophobic interactions may contribute to the adsorption process^40^.

Figure 8e compares the adsorption capacities of different cryogel structures toward AB113 under identical experimental conditions (pH 4.0, 2000 mg L^−1^ dye concentration, 30 min interaction time). The pristine Poly(HEMA-GMA) cryogel exhibits a relatively low adsorption capacity of 126.7 ± 9.8 mg g^−1^, reflecting the limited number of functional groups available for dye binding.

After modification with PLL, the adsorption capacity increases significantly to 213.9 ± 11.5 mg g^−1^ for Poly(HEMA-GMA)-PLL. This improvement can be attributed to the introduction of multiple amino groups in the PLL chains, which provide additional positively charged binding sites that interact with the anionic sulfonate groups of AB113 via electrostatic attraction.

A dramatic enhancement in adsorption performance is observed after incorporation of Cu(II) ions, where the adsorption capacity reaches 504.7 ± 16.3 mg g^−1^ for Poly(HEMA-GMA)-PLL-Cu(II). The presence of Cu(II) introduces additional coordination sites that interact with dye molecules, thereby strengthening the binding affinity of the cryogel matrix. Moreover, the combined effect of electrostatic attraction, metal–dye coordination, and improved surface functionality leads to a nearly fourfold increase in adsorption capacity compared with the unmodified cryogel.

These results clearly demonstrate that sequential functionalization with PLL and Cu(II) plays a critical role in enhancing the adsorption performance of the cryogel system, confirming the effectiveness of the designed modification strategy for efficient removal of AB113 from aqueous solutions.

**Fig. 9:**
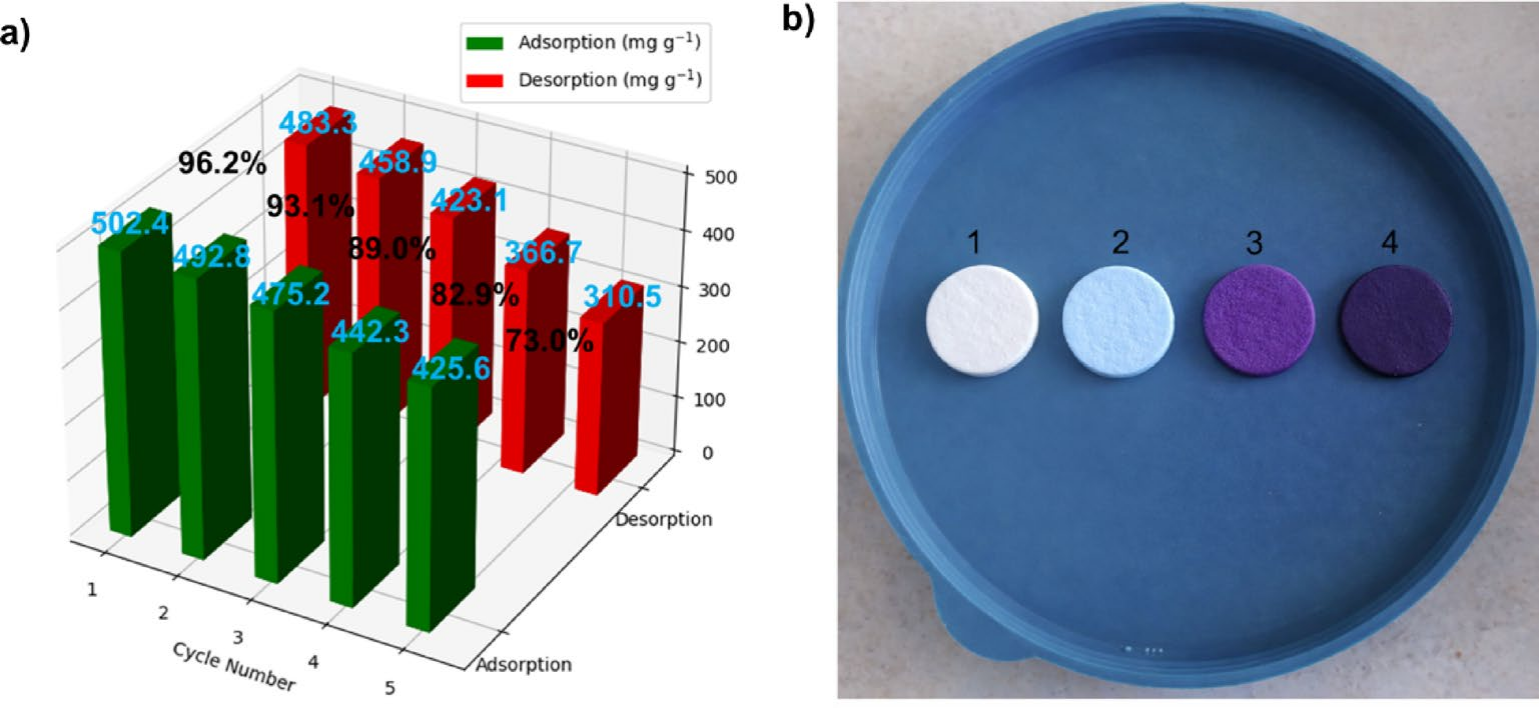
(**a**) adsorption–desorption performance over five consecutive regeneration cycles. (**b**) Photographic image of dye-loaded cryogel discs after adsorption [1: Poly(HEMA-GMA)-PLL, 2: Poly(HEMA-GMA)-PLL-Cu(II), 3: AB113 (100 mg L^− 1^) adsorbed Poly(HEMA-GMA)-PLL-Cu(II), 4: AB113 (1000 mg L^− 1^) adsorbed Poly(HEMA-GMA)-PLL-CuII)]. All cryogel discs have an average diameter of approximately 10 mm and a thickness of about 3 mm.

The reusability and regeneration ability of the Poly(HEMA-GMA)-PLL-Cu(II) cryogel were tested over five consecutive adsorption–desorption cycles. Results are shown in Fig. 9a. The cryogel initially had a high adsorption capacity of 502.4 mg g^−1^ in the first cycle, demonstrating the strong affinity of AB113 molecules for the functionalized cryogel matrix. After each regeneration, the adsorption capacity decreased gradually and moderately, reaching 425.6 mg g^−1^ after the fifth cycle. This decline is likely due to partial occupation or irreversible blockage of some active binding sites, as well as minor structural fatigue of the polymer network from repeated adsorption–desorption cycles.

Desorption capacities followed a similar trend, decreasing from 483.3 mg g^−1^ in the first cycle to 310.5 mg g^−1^ in the fifth cycle. Despite this decline, the cryogel maintained good desorption efficiency across cycles, indicating that most adsorbed dye molecules were effectively released during regeneration. The retention of relatively high adsorption capacities after multiple cycles highlights the structural stability of the macroporous cryogel architecture and the robustness of the PLL–Cu(II) coordination sites under repeated use.

Importantly, Cu(II) ions are coordinated with the multiple amino groups of the PLL chains within the cryogel matrix, forming stable metal–ligand complexes that effectively anchor the metal ions within the polymer network. This coordination structure significantly reduces the possibility of Cu(II) leaching during repeated adsorption–desorption cycles. The gradual decrease in adsorption capacity is therefore more likely associated with partial blockage of adsorption sites or incomplete desorption of dye molecules rather than the loss of Cu(II) ions from the cryogel structure.

To further evaluate the stability of the Cu(II) coordination, the regenerated solutions were visually monitored after each cycle, and no noticeable color change related to Cu species release was observed. In addition, the cryogel disc maintained its structural integrity throughout repeated adsorption–desorption cycles, indicating the mechanical and chemical stability of the cryogel matrix. These observations suggest that the Cu(II)–PLL chelation remains largely stable under the applied regeneration conditions.

These findings show that the Poly(HEMA-GMA)-PLL-Cu(II) cryogel exhibits good reusability and regenerative capacity, key factors for real-world wastewater treatment. The ability to maintain high adsorption performance across multiple cycles indicates that the developed cryogel system is a reliable, long-lasting adsorbent for effectively removing AB113 from water (Fig. 9b).

To evaluate the practical applicability of the prepared cryogel, adsorption experiments were also conducted using real wastewater. A wastewater sample collected from a local discharge source was filtered to remove suspended solids and then spiked with AB113 dye to achieve an initial concentration of 2000 mg L^−1^, which corresponds to the optimum concentration used in the batch adsorption experiments. The adsorption experiments were performed under the previously optimized conditions (pH 4.0, 10 mg adsorbent, 30 min contact time).

The results showed that the Poly(HEMA-co-GMA)-PLL-Cu(II) cryogel removed approximately 88–92% of AB113 from the real wastewater matrix, corresponding to an adsorption capacity of about 440–460 mg g^−1^. This value is slightly lower than that obtained in synthetic dye solutions (~ 500 mg g^−1^), which can be attributed to the presence of competing ions and dissolved organic matter in the wastewater matrix. Despite this small decrease, the cryogel maintained a high adsorption efficiency, demonstrating its strong affinity toward sulfonated dye molecules even in complex aqueous environments.

These results confirm that the Poly(HEMA-co-GMA)-PLL-Cu(II) cryogel retains high adsorption performance in real wastewater conditions, highlighting its potential for practical wastewater treatment applications.

###  Antibacterial and antifungal activity of cryogels

The antimicrobial activities of the synthesized copper-containing and copper-free cryogels were evaluated against a panel of Gram-positive, Gram-negative, and *Candida* species using the broth microdilution method. The MIC results clearly indicate that copper-containing cryogels exhibit markedly higher antimicrobial activity than their copper-free counterparts against all tested microorganisms (Table 1).

Notably, the copper-containing cryogels displayed pronounced activity against Gram-positive bacteria, with low MIC values of 64 µg mL^−1^ observed for *Bacillus subtilis* ATCC 6623 and *Brevibacillus brevis* ATCC 35,690. This enhanced susceptibility of Gram-positive bacteria is attributed to their relatively permeable cell walls. Although Gram-positive bacteria possess a thick peptidoglycan layer, the absence of an outer membrane facilitates the penetration of metal ions into the cell, thereby increasing their sensitivity to copper-based antimicrobial systems^41^.

In contrast, higher MIC values were generally recorded for Gram-negative bacteria. For *Enterobacter aerogenes* CCU 2531 and *Salmonella typhimurium* NRRL E 4413, the MIC values of copper-containing cryogels reached 512 µg mL^−1^. This reduced susceptibility is commonly associated with the presence of an outer membrane in Gram-negative bacteria, which acts as an effective permeability barrier against metal ions and other antimicrobial agents^42^. Consistent with this observation, previous studies have reported that copper and copper-based materials typically exhibit lower antimicrobial efficacy against Gram-negative bacteria compared to Gram-positive strains^43^.

Regarding fungal strains, the copper-containing cryogels demonstrated detectable antifungal activity against *Candida glabrata* ATCC 15,126 and *Candida tropicalis* ATCC 13,803; however, relatively high MIC values (1024 µg mL^−1^) were observed. The limited antifungal efficacy may be explained by the eukaryotic nature of *Candida* species, which possess more complex cellular structures and ergosterol-rich membranes that confer increased resistance to metal ions^44^. Importantly, the complete absence of antifungal activity in copper-free cryogels confirms that the observed antifungal effect originates directly from the presence of copper within the cryogel matrix.

Compared with the reference antibiotics used in this study, namely amoxicillin and tetracycline, the cryogels exhibited substantially higher MIC values against the bacterial strains. However, it should be emphasized that the cryogels are not intended to function as conventional antibiotics. Rather, they are evaluated as alternative antimicrobial materials. In this context, the moderate yet broad-spectrum antimicrobial activity observed for copper-containing cryogels is noteworthy. Such materials may be particularly advantageous as supportive antimicrobial systems, for example, in antimicrobial surfaces, wound dressings, and other biomedical or environmental applications where sustained activity and material stability are required^45^.

The MIC data demonstrate the antimicrobial efficacy of copper-containing cryogels and highlight the critical role of copper in imparting biological activity. These findings contribute to the growing body of literature on metal-based polymeric systems and support their potential as multifunctional materials combining adsorption capability with intrinsic antimicrobial properties.

**Table 1:** MIC values (µg mL^− 1^) of the tested molecules.

	A	B	C	D	E	F	G	H
With copper	64	64	512	32	512	512	1024	1024
Without copper	512	512	1024	512	1024	1024	-	-
Amoxicillin	< 2	< 2	< 1	> 32	> 1024	> 1024	ND	ND
Tetracycline	< 2	< 2	< 2	2	< 2	> 4	ND	ND

With copper: Poly(HEMA-GMA)-PLL-Cu(II), Without copper: Poly(HEMA-GMA)-PLL, A: *Bacillus subtilis* ATCC 6623, B: *Brevibacillus brevis* ATCC 35,690, C: *Lysteria monocytogenes* NCTC 5348, D: *Escherichia coli* ATCC 25,922, E: *Enterobacter aerogenes* CCU 2531, F: *Salmonella typhimurium* NRRLE 4413, G: *Candida glabrata* ATCC 15,126, H: *Candida tropicalis* ATCC 13,803.

### Adsorption behavior and mechanistic insights

 The equilibrium adsorption data obtained at room temperature were best described by the Langmuir isotherm, as evidenced by the highest correlation coefficient among the tested models (Table 2). This indicates that AB113 adsorption proceeds predominantly via monolayer coverage on a set of energetically uniform active sites within the cryogel structure. The high maximum adsorption capacity (q_max_​= 512.8 mg g^−1^) highlights the strong affinity of the Poly(HEMA-GMA)-PLL-Cu(II) cryogel toward AB113 molecules.

The Freundlich model also provided a reasonable fit, with an nnn value greater than unity, suggesting favorable adsorption and moderate surface heterogeneity. This behavior implies that, while monolayer adsorption dominates, the cryogel surface still contains sites with varying adsorption energies. The Temkin model further supports this interpretation by showing a gradual decrease in adsorption energy with increasing surface coverage, consistent with adsorbate–adsorbate interactions becoming more significant at higher concentrations.

The D–R isotherm yielded a mean adsorption energy below 8 kJ mol^−1^, confirming that the adsorption process is mainly governed by physical interactions such as electrostatic attraction, hydrogen bonding, and diffusion within the macroporous cryogel network^46^. Collectively, these results demonstrate that AB113 adsorption on the Poly(HEMA-GMA)-PLL-Cu(II) cryogel is a highly efficient, predominantly physisorption-controlled process occurring on a structurally well-defined adsorption surface.

**Table 2:** Isotherm model parameters.

Model	Parameters (value)	*R* ^2^
Langmuir	q_max_=512.8 mg g^−1^; K_L_=0.0146 L mg^−1^	0.998
Freundlich	K_F_=96.4 (mg g^−1^)(L mg^−1^)^1/n^; 1/*n* = 0.31; *n* = 3.23	0.944
Temkin	K_T_=0.82 L mg^−1^; B_T_=68.7 J mol^−1^	0.962
D–R	q_m_=498.6 mg g^−1^; β = 1.9 × 10^−8^ mol^2^ J^−2^; E = 5.1 kJ mol^−1^	0.931

The adsorption behavior of AB113 onto the Poly(HEMA-GMA)-PLL-Cu(II) cryogel was systematically evaluated at 10, 30, and 50 °C under acidic conditions (pH 4.0) and a high initial dye concentration (2000 mg L^−1^). The time-dependent uptake profiles reveal a rapid adsorption phase within the first 20–30 min, followed by a pronounced plateau, indicating rapid occupation of available binding sites and the subsequent establishment of adsorption equilibrium. This rapid kinetic response can be attributed to the highly interconnected macroporous structure of the cryogel, which facilitates efficient mass transport of AB113 molecules toward the interior binding domains (Table 3a).

The kinetic data are best described by the PSO model at all investigated temperatures, as evidenced by the excellent correlation coefficients (R^2^ ≈ 0.998–0.999) and the close agreement between experimental and calculated equilibrium adsorption capacities (Table 3b). The superiority of the PSO model suggests that the overall adsorption rate is controlled by the availability and reactivity of active sites on the cryogel surface rather than by external diffusion limitations. In this system, these active sites are primarily associated with Cu(II) coordination centers and protonated amine groups of PLL, which can strongly interact with the sulfonate groups of AB113 through electrostatic attraction and metal–dye coordination.

Temperature exerts a pronounced influence on both the adsorption capacity and the kinetic rate constants. At 10 and 30 °C, nearly identical equilibrium capacities of approximately 502 mg g^−1^ are achieved, indicating that the adsorption process is highly efficient and thermodynamically favorable under mild conditions. In contrast, increasing the temperature to 50 °C leads to a notable decrease in equilibrium capacity (404.7 mg g^−1^) and a reduction in the PSO rate constant. This behavior implies partial destabilization of dye–adsorbent interactions at elevated temperatures, which is characteristic of exothermic adsorption processes dominated by physical interactions and reversible coordination bonding.

The thermodynamic analysis further supports this interpretation. The ΔG° values at all temperatures confirm the spontaneous nature of AB113 adsorption onto the cryogel. However, the gradual increase in ΔG° (less negative) with rising temperature indicates a decreasing driving force for adsorption, consistent with the observed reduction in uptake at 50 °C. The negative ΔH° confirms that the adsorption process is exothermic, while its moderate magnitude suggests that AB113 removal is governed primarily by physical interactions, including electrostatic attraction, hydrogen bonding, and Cu(II)–dye coordination, rather than by strong chemisorption. Additionally, the negative ΔS° reflects a decrease in randomness at the solid–liquid interface, which can be attributed to the ordered arrangement of dye molecules upon binding to the structured cryogel network (Table 3c).

Taken together, the combined kinetic and thermodynamic results demonstrate that Poly(HEMA-GMA)-PLL-Cu(II) cryogels offer rapid, efficient, and spontaneous adsorption of AB113 under mild temperature conditions. The strong performance at low to ambient temperatures, coupled with the short equilibrium time, highlights the practical potential of this system for high-concentration dye removal applications, particularly in energy-efficient wastewater treatment processes where operation at elevated temperatures is undesirable.

**Table 3:** Integrated kinetic and thermodynamic analysis. Experimental conditions: pH = 4.0, initial AB113 concentration = 2000 mg L^−1^.

(a) Experimental kinetic data							
Time (min)		10 °C q_t_ (mg g^−1^)	30 °C q_t_ (mg g^−1^)	50 °C q_t_ (mg g^−1^)			
0		0	0	0			
5		255.2	260.5	225.0			
10		325.6	327.8	305.2			
20		435.8	430.9	385.6			
30		501.3	501.6	402.0			
45		501.6	501.8	403.2			
60		501.8	502.0	404.0			
90		502.4	502.5	404.7			

(b) Kinetic model parameters							
Temp (°C)	qₑ,exp		k_1_	PFO *R*^2^	k_2_	q_e_,cal	PSO *R*^2^
10	502.4		0.061	0.93	0.00023	504.1	0.999
30	502.5		0.064	0.94	0.00026	505.0	0.999
50	404.7		0.048	0.91	0.00014	406.8	0.998

(c) Thermodynamic parameters							
Temp (°C)	T (K)		ΔG° (kJ mol^−1^)				
10	283		−6.8				
30	303		−6.2				
50	323		−5.1				

ΔH° = −18.4 kJ mol^−1^, ΔS° = −41.2 J mol^−1^ K^−1^.

### Literature review

Table 4 provides a comparative overview of AB113 adsorption performances of different adsorbent systems, highlighting the adsorption capacity, operating conditions, interaction time, and regeneration behavior. The Poly(HEMA-GMA)-PLL-Cu(II) cryogel developed in this study exhibits a high adsorption capacity of 501.6 mg g^−1^ at a relatively short optimum interaction time of 30 min under acidic conditions (pH 4.0) and a high initial dye concentration (2000 mg L^−1^). Compared to chitosan-based composites and fibrous membranes, this rapid uptake represents a clear kinetic advantage, as most literature systems require long-term equilibration periods (up to 120 h) to reach their maximum capacities^47–50^.

Although some chitosan-derived materials report higher maximum adsorption capacities^48–50^, these values are typically achieved at much lower initial dye concentrations and after prolonged contact times, which limits their practical applicability in continuous or high-throughput treatment processes. In contrast, the cryogel system demonstrates a balanced performance, combining high capacity, fast kinetics, and operation at realistic high pollutant loads.

Regeneration studies further indicate that the cryogel can be efficiently regenerated using a mixed NaCl/ethanol alkaline desorption medium, retaining substantial adsorption capacity over five consecutive cycles despite a gradual decrease from 502.4 to 425.6 mg g^−1^. This reusability is comparable to or better than several reported systems, some of which either lack regeneration data or show significant capacity losses within fewer cycles. Overall, the comparison underscores that the Poly(HEMA-GMA)-PLL-Cu(II) cryogel offers a competitive and practically attractive alternative, particularly when fast adsorption, high working concentration, and repeated use are prioritized over maximum theoretical capacity.

**Table 4:** Comparative evaluation of AB113 adsorption performance.

Adsorbent	Optimum conditions	Adsorption capacity (mg g^−1^)	Optimum interaction time	Desorption/regeneration method	Reusability	Ref
Poly(HEMA-GMA)-PLL-Cu(II) cryogel	pH 4.0; C₀ = 2000 mg L^−1^	501.6	30 min	0.5 M NaCl + 30% (v/v) ethanol; pH 10; 60 min; 25 °C	5 cycles: capacity decreased from 502.4 to 425.6 mg g^−1^	This study
C–Fe_2_O_3_ (chitosan-based composite)	pH 3.0; C₀ = 10 mg L^−1^; adsorbent dose = 0.6 g L^−1^	124.2	120 min	Ethanol and deionized water	6 cycles: only a slight loss in efficiency	^47^
Pure chitosan nanofibrous membrane	Concentration range: 50–250 mg L^−1^; long-term kinetic equilibrium	1309.0	120 h	0.1 M NaOH	4 cycles: ~70% capacity retention (596.6 mg g^−1^ after 4th cycle)	^48^
Micro–nanofibrous chitosan sponge	Long-term adsorption experiments	604.7	120 h	Not reported	Not reported	^49^
CESA composite sponge	pH 3.0; C₀ = 100 mg L^−1^; adsorbent dose = 10 mg	926.2	120 h	0.05 M NaOH (ethanol/water = 1:2, v/v); 10 min	4 cycles: only 11% loss in efficiency	^50^

C_o_: Initial AB113 concentration, CESA: Chitosan/electrospun SA nanofiber composite sponges.

## Conclusion

This study demonstrates the successful development of a Poly(HEMA-GMA)-PLL-Cu(II) cryogel as an effective adsorbent for the removal of the anionic dye Acid Blue 113 from aqueous media. The integration of a macroporous cryogel structure with PLL functionalization and Cu(II) coordination created a multifunctional adsorption platform capable of promoting rapid mass transfer and multiple interaction mechanisms. The combined presence of protonated amine groups and metal-coordination sites enabled strong electrostatic and coordination-assisted interactions with the dye molecules, resulting in high adsorption efficiency.

The adsorption experiments revealed that the cryogel possesses a high adsorption capacity exceeding 500 mg g^−1^ and reaches equilibrium within a short interaction time of 30 min, highlighting the advantages of the interconnected macroporous structure. The adsorption process was well described by the Langmuir isotherm and pseudo-second-order kinetic model, while thermodynamic analysis confirmed that the adsorption of AB113 is spontaneous and exothermic.

In addition to its adsorption capability, the Cu(II)-containing cryogel exhibited antibacterial activity against several microorganisms, suggesting an additional benefit for water treatment systems where microbial growth and biofouling may occur. Furthermore, the cryogel maintained considerable adsorption performance after multiple adsorption–desorption cycles, demonstrating good structural stability and regeneration potential.

These findings highlight the potential of Poly(HEMA-GMA)-PLL-Cu(II) cryogels as multifunctional materials that combine high adsorption capacity, rapid adsorption kinetics, antimicrobial functionality, and reusability, making them promising candidates for advanced treatment of dye-contaminated wastewater.

## Supplementary Information

Below is the link to the electronic supplementary material.

Supplementary Material 1

## Data Availability

The datasets used and/or analyzed during the current study are available from the corresponding author upon reasonable request.
