# Supplementary material for: Amine-functionalized copper-coordinated cryogels for efficient removal of Acid Blue 113 from water
Source: Sci Rep. 2026 Apr 12;16:17013. doi: 10.1038/s41598-026-47887-8 (PMC13230942; doi:10.1038/s41598-026-47887-8)
Supplement: Supplementary file 1 — Supplementary Material 1 [file 41598_2026_47887_MOESM1_ESM.docx]

**Table S1.** Adsorption isotherm models used for equilibrium analysis.

| **Isotherm model** | **Non-linear form** | **Linearized form** | **Parameters** |
| --- | --- | --- | --- |
| Langmuir | q_e_ = (q_max_ K_L_ C_e_) / (1 + K_L_ C_e_) | C_e_ / q_e_ = 1/(q_max_ K_L_) + C_e_ / q_max_ | q_max_ (mg.g⁻¹): maximum monolayer capacity; K_L_ (L.mg⁻¹): Langmuir constant |
| Freundlich | q_e_ = K_F_ C_e_^(1/n)^ | ln qe = ln K_F_ + (1/n) ln C_e_ | K_F_: adsorption capacity constant; n: adsorption intensity |
| Dubinin–Radushkevich (D–R) | q_e_ = q_m_ exp(−β ε²) | ln q_e_ = ln q_m_ − β ε² | q_m_ (mg.g⁻¹): theoretical capacity; β: D–R constant;  E = (2β)^−1/2^ |
| Temkin | q_e_ = B_T_ ln(K_T_ C_e_) | q_e_ = B_T_ ln K_T_ + B_T_ ln C_e_ | B_T_ = RT/b; K_T_ (L.mg⁻¹): Temkin constant; b (J.mol⁻¹) |

Note: q_e_ (mg.g⁻¹) and C_e_ (mg.L⁻¹) denote equilibrium adsorption capacity and concentration, respectively. For the D–R model, ε = RT ln(1 + 1/C_e_).

The following equation describes the pseudo-first-order (PFO) kinetic model:

$\ln{(q}_{e}-q_{t}$) = $\ln q_{e}$- $k_{1}$.t (Eq 1)

Where q_e_​ (mg g^−1^) and q_t_ (mg g^−1^) represent the adsorption capacities at equilibrium and at time t (min), respectively, and k_1_​ (min^−1^) is the pseudo-first-order rate constant.

The pseudo-second-order (PSO) kinetic model is expressed as:

$\frac{t}{q_{t}}$ = $\frac{1}{k_{2}{q_{e}}^{2}}+ \frac{t}{q_{t}}$ (Eq 2)

where k_2_​ (g mg^−1^ min^−1^) denotes the pseudo-second-order rate constant.

The kinetic parameters for both models were obtained from the linearized forms of the corresponding equations. The applicability of each kinetic model was evaluated based on correlation coefficients (R²) and on the agreement between the experimentally determined and model-predicted equilibrium adsorption capacities (q_e_).

**Table S2.** Thermodynamic equations and parameters for AB113 adsorption.

| **Parameter** | **Equation** |
| --- | --- |
| Distribution coefficient | K_d_ = q_e_ / C_e_  (Eq 3) |
| Dimensionless equilibrium constant | K = 1000 × K_d_ (Eq 4) |
| Gibbs free energy change | ΔG° = −RT ln K (Eq 5) |
| Van’t Hoff equation | ln K = (ΔS° / R) − (ΔH° / RT) (Eq 6) |

**Table S3.** BET-derived pore structure parameters of the synthesized cryogels, including specific surface area, total pore volume, and average pore diameter for Poly(HEMA-GMA), Poly(HEMA-GMA)-PLL, and Poly(HEMA-GMA)-PLL-Cu(II).

| **Cryogel** | **Surface area (m² g⁻¹)** | **Pore volume (cm³ g⁻¹)** | **Average pore diameter (nm)** |
| --- | --- | --- | --- |
| Poly(HEMA-GMA) | 6.45 | 0.021 | 36 |
| Poly(HEMA-GMA)-PLL | 7.12 | 0.024 | 38 |
| Poly(HEMA-GMA)-PLL-Cu(II) | 7.85 | 0.028 | 41 |
